# Effect of a structured tracheostomy educational programme on healthcare professionals’ knowledge and confidence: A quasi-experimental pre-test–post-test study at Nelson Mandela Academic Hospital

**DOI:** 10.4102/jcmsa.v4i1.236

**Published:** 2026-09-17

**Authors:** Zanele Tuswa, Banele Kokose, Mirabel K.K. Nanjoh, Charles Myataza

**Affiliations:** 1 Department of Surgery for Ear, Nose and Throat, Faculty of Health Sciences, Walter Sisulu University, Mthatha, South Africa; 2 Department of Public Health, Faculty of Health Sciences, Walter Sisulu University, Mthatha, South Africa

**Keywords:** tracheostomy, tracheostomy care, tracheostomy knowledge, tracheostomy indications and complications, coronavirus disease 2019 (COVID-19), tracheostomy care

## Abstract

**Background:**

Tracheostomy care requires healthcare professionals to recognise and manage routine and emergency complications promptly. Knowledge and preparedness gaps may compromise patient safety, particularly in resource-limited settings. This study assessed baseline knowledge and self-reported confidence in tracheostomy care and evaluated the immediate effect of a structured educational programme at Nelson Mandela Academic Hospital.

**Methods:**

A quantitative quasi-experimental one-group pre-test–post-test study was conducted among healthcare professionals involved in tracheostomy care. Participants completed structured questionnaires before and immediately after a tracheostomy educational programme. Changes in knowledge and confidence were analysed using McNemar’s test, Friedman’s test and generalised estimating equations (GEE).

**Results:**

Seventy-seven healthcare professionals participated. High overall knowledge increased from 14.3% pre-intervention to 67.5% post-intervention (*p* < 0.001). Participants had significantly higher odds of adequate knowledge after the intervention (adjusted OR 11.71, 95% CI 4.30–31.88; *p* < 0.001), while > 11 years of clinical experience was also associated with adequate knowledge (adjusted OR 4.74, 95% CI 1.77–12.69; *p* = 0.002). Confidence in tracheostomy tube insertion improved significantly (*p* = 0.003), with participants aged ≤ 40 years more likely to report high confidence (adjusted OR 2.27, 95% CI 1.06–4.88; *p* = 0.035).

**Conclusion:**

A structured tracheostomy educational programme significantly improved healthcare professionals’ knowledge and self-reported confidence. Regular education incorporating simulation may improve preparedness and promote safer tracheostomy care, particularly in resource-limited settings.

**Contribution:**

This study contributes to the limited South African literature on healthcare professionals’ knowledge of tracheostomy care by identifying important knowledge gaps and demonstrating that a structured educational intervention can significantly improve both knowledge and confidence. The findings provide locally relevant evidence to support the development of standardised tracheostomy-care training, regular refresher programmes and institutional protocols aimed at improving the safety and quality of tracheostomy care.

## Introduction

Tracheostomy remains a common and potentially life-saving airway intervention requiring coordinated care by appropriately trained healthcare professionals. Despite advances in tracheostomy techniques and postoperative care, tracheostomy-related complications continue to occur, highlighting the importance of adequate knowledge, adherence to care protocols and preparedness to recognise and manage airway emergencies.^1,2,3,4,5^ Safe tracheostomy care therefore requires healthcare professionals to understand established principles of routine care and the recognition and management of potentially life-threatening complications.^2,4,5^

Studies from Pakistan, Ethiopia, Saudi Arabia, Türkiye, India and Sudan have identified important gaps in healthcare professionals’ knowledge and preparedness regarding routine and emergency tracheostomy care.^6,7,8,9,10,11^ Structured educational interventions have been associated with improvements in tracheostomy-related knowledge and confidence.^11,12,13^ Simulation-based education, including low-cost part-task trainers, has become an increasingly important component of tracheostomy education. Bonduelle et al. demonstrated that a low-cost paediatric tracheostomy trainer can provide an effective and accessible method for procedural simulation, particularly where access to commercial simulators is limited.^14^ However, there is limited evidence from South African tertiary healthcare settings regarding healthcare professionals’ knowledge of tracheostomy care and the immediate effects of structured educational interventions addressing these gaps.^15^

Paediatric tracheostomy care presents additional challenges because of the smaller airway, the potential for accidental tube dislodgement and the need for rapid recognition and management of airway emergencies. Pritchett et al. demonstrated variability in nurses’ and caregivers’ comfort in managing paediatric tracheostomy care and emergencies, highlighting the importance of structured education and preparedness in this high-risk area of care.^16^ The inclusion of a paediatric tracheostomy simulation component in the present educational programme was intended to provide practical demonstration of principles relevant to emergency paediatric tracheostomy tube replacement.

This study therefore aimed to assess baseline tracheostomy care knowledge and self-reported confidence among healthcare professionals and evaluate the immediate effect of a structured tracheostomy educational programme at a South African tertiary academic hospital.

## Research methods and design

### Study design

A quantitative, quasi-experimental one-group pre-test–post-test study was conducted to evaluate the effect of a structured tracheostomy educational programme on healthcare professionals’ knowledge and self-reported confidence regarding routine and emergency tracheostomy care. The educational intervention was conducted at the Mthatha Health Resource Centre on 24 October 2024 and 25 October 2024. Each workshop lasted approximately 3 h and consisted of a pre-intervention questionnaire, a structured tracheostomy educational programme, a post-intervention questionnaire, a facilitated discussion and a question-and-answer session.

### Participants

Healthcare professionals working in the adult, paediatric, and neonatal intensive care units, surgical wards, medical wards, trauma units, and high-care areas at Nelson Mandela Academic Hospital were invited to participate. Participants were recruited using convenience sampling. Healthcare professionals who completed both the pre-intervention and post-intervention questionnaires were included in the final analysis, whereas those who did not complete either assessment were excluded.

### Questionnaire

Data were collected using a structured self-administered questionnaire adapted from the validated questionnaire developed by Khanum et al., with modifications to align with the objectives of the present study.^6^ The original questionnaire was designed to assess healthcare professionals’ knowledge regarding tracheostomy care and the management of early tracheostomy-related complications. The questionnaire was adapted to align with the objectives of the present study and current evidence-based recommendations for tracheostomy care. It comprised two sections: demographic and professional characteristics, and questions assessing knowledge of tracheostomy indications, bedside care, complications and self-reported confidence in tracheostomy tube insertion.

### Educational intervention

The structured tracheostomy educational programme was developed and delivered by an otorhinolaryngologist with fellowship training in facial plastic and reconstructive surgery. The programme combined didactic teaching with practical demonstrations to improve participants’ knowledge and self-reported confidence in routine and emergency tracheostomy care. The educational programme was designed in accordance with internationally recognised principles of safe tracheostomy care, including recommendations from the Global Tracheostomy Collaborative (GTC), the National Tracheostomy Safety Project (NTSP) and multidisciplinary guidance for tracheostomy emergencies. Core components included routine bedside care, emergency airway management, multidisciplinary communication and standardised approaches to accidental decannulation and tube obstruction.

The practical demonstration included hands-on familiarisation with different tracheostomy tubes, including cuffed and uncuffed tubes, as well as single-lumen and double-lumen tubes. Participants were shown the design features, indications and appropriate clinical applications of each tube type to enhance understanding of tracheostomy equipment used in clinical practice.

A low-cost paediatric tracheostomy simulation model was developed using a modified plastic bottle to demonstrate paediatric tracheostomy tube replacement. The simulation model was developed as an educational demonstration tool to illustrate the principles of emergency paediatric tracheostomy tube replacement in a resource-constrained setting. It was not subjected to formal face, content or construct validation prior to implementation and was not intended to replace high-fidelity simulation. Rather, it was designed as a low-cost teaching adjunct to reinforce key procedural concepts during the educational workshop. A vertical incision represented the tracheostomy, while stay sutures were attached to either side of the simulated tracheal opening and labelled ‘left’ and ‘right’ to demonstrate correct identification and handling during emergency tube replacement. Participants were shown how gentle lateral traction on the stay sutures opens the tracheostomy to facilitate safe reinsertion of the tracheostomy tube while maintaining correct orientation of the sutures. An intraoperative clinical photograph demonstrating correctly labelled paediatric tracheal stay sutures was also presented to reinforce the principles demonstrated using the simulation model.

The educational programme concluded with a facilitated discussion and question-and-answer session, allowing participants to clarify concepts and discuss common clinical scenarios encountered during routine and emergency tracheostomy care.

### Statistics

Data were entered into Microsoft Excel (Microsoft Corporation, Redmond, WA, United States) and analysed using Stata version 17 (Stata Corp LLC, College Station, TX, United States). Descriptive statistics were used to summarise participants’ demographic and professional characteristics. Categorical variables were presented as frequencies and percentages. Changes in paired categorical responses between the pre-intervention and post-intervention assessments were analysed using McNemar’s test. Changes in overall knowledge and confidence levels were assessed using Friedman’s test.

Generalised estimating equations (GEE) with a logit link function and exchangeable correlation structure were used to identify factors independently associated with adequate knowledge and high confidence while accounting for repeated measurements obtained from the same participants. Variables entered into the multivariable models included study time (pre-intervention versus post-intervention), age group, gender, professional category, hospital department, years of clinical experience, knowledge level and confidence level where appropriate. Adjusted odds ratios (aORs) with 95% confidence intervals (CIs) were reported as measures of effect size for the multivariable GEE analyses. Statistical significance was set at a two-sided *p*-value < 0.05.

### Ethical considerations

Ethical clearance to conduct this study was obtained from Walter Sisulu University and Health Science Research Ethics Committee (No. REC-120209-020). Permission to conduct the study was granted by the Eastern Cape Department of Health and the management of Nelson Mandela Academic Hospital. Written informed consent was obtained from all participants before enrolment. Participation was voluntary, and participants were informed of their right to withdraw from the study at any stage without penalty. To ensure confidentiality, questionnaires were anonymised, and no personally identifiable information was collected. All study data were stored securely and were accessible only to the research team.

## Results

A total of 77 healthcare professionals completed both the pre-intervention and post-intervention assessments and were included in the final analysis. Table 1 summarises the demographic and professional characteristics of the participants. Medical officers and registrars constituted the largest professional group (42.9%), followed by professional nurses (31.2%), enrolled nursing assistants (16.9%) and specialists (9.1%). Most participants were older than 30 years (84.5%), worked in surgical wards (45.5%) and had more than 11 years of clinical experience (39.0%).

**TABLE 1:** Demographic and professional characteristics of the participants (*N* = 77).

Category	Variable	*n*	%
Gender	Female	50	64.90
	Male	27	35.00
Age group (years)	26–30	5	6.50
	31–40	32	41.60
	41–50	24	31.20
	Above 51	9	11.70
	Less than 25	7	9.10
Occupation	Specialist	7	9.10
	Enrolled nursing assistant	13	16.90
	Medical Officer/Registrar	33	42.90
	Professional nurse	24	31.20
Hospital department	Adult ICU	10	13.00
	Medical ward	8	10.40
	Paediatric/neonatal ICU/high care	11	14.30
	Surgical ward	35	45.50
	Trauma ward/high care	13	16.90
Experience (years)	3–5	12	15.60
	5–8	10	13.00
	8–11	12	15.60
	Less than 2	13	16.90
	Over 11	30	39.00

ICU, intensive care unit.

Table 2 presents participants’ knowledge regarding tracheostomy indications, bedside care and complications before and after the structured tracheostomy educational programme. Knowledge of tracheostomy indications improved significantly following the intervention, with the proportion of correct responses increasing from 63.6% to 83.1% (McNemar, *p* = 0.003). Significant improvements were observed across nearly all domains of bedside tracheostomy care. The greatest improvement was observed in recognition of the first sign of a blocked tracheostomy tube, where correct responses increased from 36.4% to 83.1%, followed by knowledge of the recommended suctioning interval for established tracheostomies, which improved from 46.8% to 90.9%. The smallest improvement was observed for prevention of stomal infection, where baseline knowledge was already high (76.6% vs. 85.7%), and the difference did not reach statistical significance (*p* = 0.095). Knowledge of tracheostomy-related complications also improved significantly following the educational programme, with correct responses increasing from 14.3% before the intervention to 42.9% afterwards (McNemar, *p* < 0.001).

**TABLE 2:** Pre- and post-intervention knowledge distribution of tracheostomy indication, bedside care and complications (*N* = 77).

Knowledge domain	Study time								McNemar *p*-value
	Pre-lecture				Post-lecture				
	Incorrect		Correct		Incorrect		Correct		
	*n*	%	*n*	%	*n*	%	*n*	%	
**Knowledge of the indication**									
Know the indications of tracheostomy	28	36.4	49	63.6	13	16.9	64	83.1	0.0030
**Knowledge of bedside tracheostomy care**									
Ideal time interval for tracheostomy suctioning (early tracheostomy)	20	26.0	57	74.0	1	1.3	76	98.7	< 0.0001
Ideal time interval for tracheostomy suctioning (late tracheostomy)	41	53.2	36	46.8	7	9.1	70	90.9	< 0.0001
First sign of a blocked tracheostomy tube	49	63.6	28	36.4	13	16.9	64	83.1	< 0.0001
Preventing tracheostomy tube dislodgement	31	40.3	46	59.7	7	9.1	70	90.9	< 0.0001
Purpose of saline instillation before suctioning into the tracheostomy tube	22	28.6	55	71.4	6	7.8	71	92.2	< 0.0001
How to prevent stomal infection	18	23.4	59	76.6	11	14.3	66	85.7	0.0950
How deep must one insert the suction tube into the tracheostomy tube during suctioning	31	40.3	46	59.7	11	14.3	66	85.7	< 0.0001
In case of a completely blocked tracheostomy	25	32.5	52	67.5	12	15.6	65	84.4	0.0040
The safest way for tracheostomy care in a COVID-19 patient	38	49.4	39	50.6	25	32.5	52	67.5	0.0180
**Knowledge of complications**									
Know the complications of tracheostomy	66	85.7	11	14.3	44	57.1	33	42.9	< 0.0010

Overall knowledge scores improved significantly following the structured tracheostomy educational programme (Table 3). The proportion of participants classified as having high knowledge increased from 14.3% before the intervention to 67.5% afterwards, while the proportion with low knowledge decreased from 36.4% to 6.5% (Friedman test, *p* < 0.001).

**TABLE 3:** Total knowledge scores and overall knowledge level of tracheostomy indications, care and complications pre- and post-intervention (*N* = 77).

Overall knowledge level	Pre-lecture		Post-lecture	
	*n*	%	*n*	%
**Knowledge category**				
Low	28	36.4	5	6.5
Moderate	38	49.4	20	26.0
High	11	14.3	52	67.5

After adjustment for potential confounders using GEE, study time and years of clinical experience remained independently associated with adequate knowledge (Table 4). Participants assessed after the educational programme had significantly higher odds of demonstrating adequate knowledge than before the intervention (adjusted OR 11.71, 95% CI 4.30–31.88; *p* < 0.001). Participants with more than 11 years of clinical experience were also significantly more likely to demonstrate adequate knowledge than those with 11 years or less (adjusted OR 4.74, 95% CI 1.77–12.69; *p* = 0.002). No significant associations were observed for age, gender, professional category, hospital department or baseline confidence level.

**TABLE 4:** Socio-demographic influence on tracheostomy knowledge.

Predictors	Regression coefficient (B)	Standard error	Wald *χ*^2^	Adjusted odds ratio	95% CI	*p*-value
**Study time**						
Post-lecture	2.460	0.5110	23.181	11.71	4.30–31.88	< 0.001
Pre-lecture	-	-	-	Reference	-	-
**Gender**						
Male	0.973	0.5711	2.906	2.65	0.86–8.11	0.088
Female	-	-	-	Reference	-	-
**Department**						
Surgical ward	−0.072	0.5933	0.015	0.93	0.29–2.98	0.903
Medical ward	0.256	0.9018	0.080	1.291	0.22–7.56	0.777
ICU/Trauma/High care	-	-	-	Reference	-	-
**Age group (years)**						
≤ 40	0.820	0.5198	2.490	2.27	0.82–6.29	0.115
> 40	-	-	-	Reference		-
**Professional category**						
Nursing	−0.682	0.5635	1.465	0.51	0.17–1.53	0.226
Medicine	-	-	-	Reference	-	-
**Years working**						
Over 11	1.556	0.5025	9.587	4.74	1.77–12.69	0.002
Up to 11	-	-	-	Reference	-	-
**Confidence level**						
Low	−0.346	0.4371	0.628	0.71	0.30–1.67	0.428
High	-	-	-	Reference	-	-
**Intercept**	−0.456	0.7197	0.402	0.63	0.16–2.60	0.526

ICU, intensive care unit; CI, confidence interval.

Participants’ self-reported confidence in tracheostomy tube insertion improved significantly following the structured tracheostomy educational programme (Figure 1). Before the intervention, responses were predominantly clustered within the no, low and moderate confidence categories, whereas after the intervention, there was a significant shift towards the confident and highly confident categories (Friedman test, *p* = 0.003).

**FIGURE 1:**
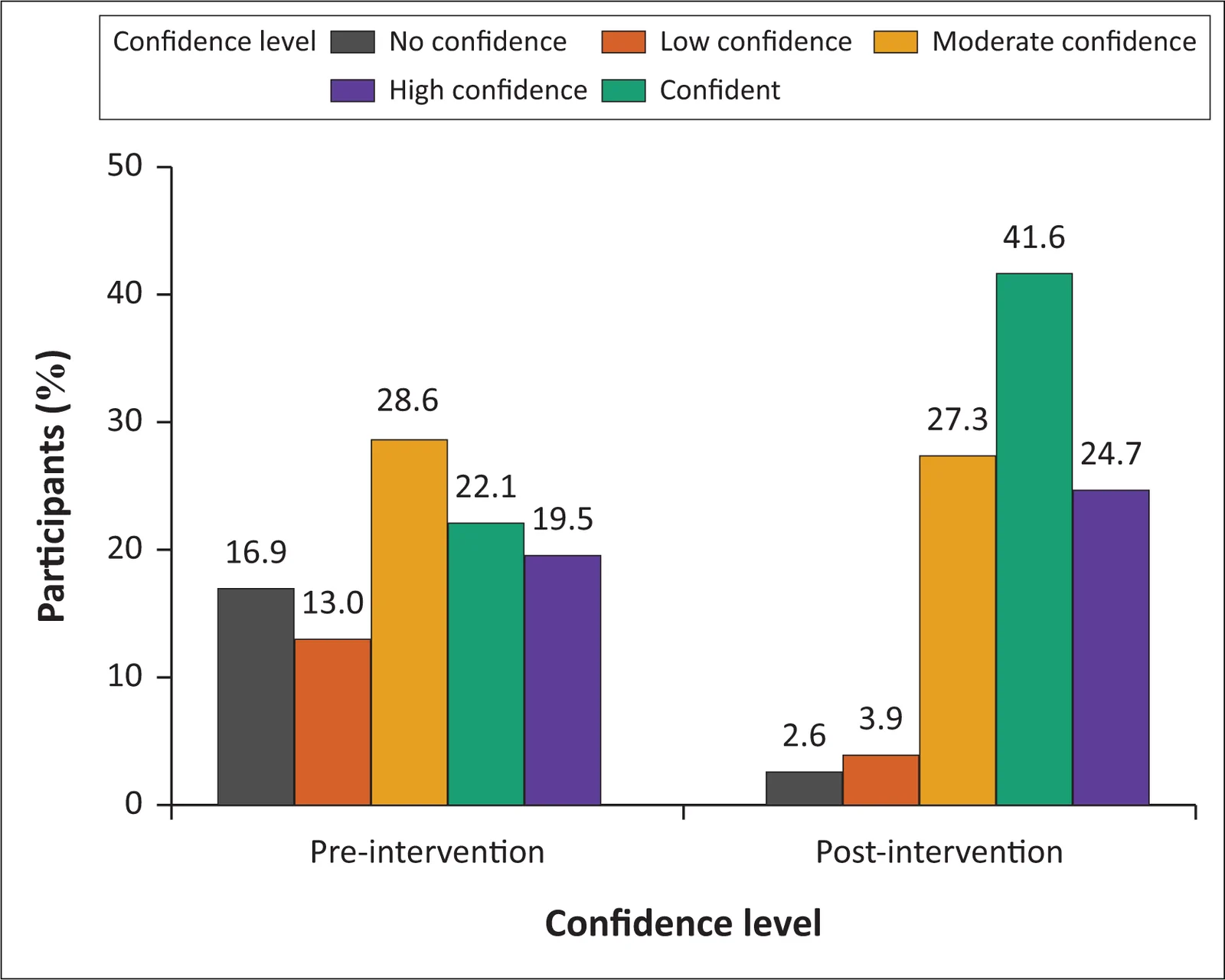
Level of confidence in inserting the tracheostomy tube.

Multivariable GEE analysis demonstrated that participants assessed after the educational programme were significantly more likely to report high confidence than before the intervention (adjusted OR 3.43, 95% CI 1.62–7.29; *p* = 0.001). Participants aged 40 years or younger also had significantly greater odds of reporting high confidence than those older than 40 years (adjusted OR 2.27, 95% CI 1.06–4.88; *p* = 0.035) (Table 5). Gender, professional category, hospital department, years of experience and knowledge level were not independently associated with confidence.

**TABLE 5:** Socio-demographic influence on confidence in inserting a tracheostomy tube.

Predictors of confidence level to insertion of a tracheostomy tube	Regression coefficient (B)	Standard error	Wald *χ*^2^	Adjusted odds ratio	95% CI	*p*-value
**Study time**						
Post-lecture	1.233	0.3845	10.279	3.43	1.62–7.29	0.001
Pre-lecture	-	-	-	Reference	-	-
**Gender**						
Male	−0.044	0.3502	0.016	1.00	0.48–1.90	0.899
Female	-	-	-	Reference	-	-
**Department**						
Surgical ward	0.624	0.4072	2.345	1.87	0.84–4.14	0.126
Medical ward	0.837	0.7134	1.377	2.31	0.57–9.35	0.241
ICU/Trauma/High care	-	-	-	Reference	-	-
**Age group (years)**						
≤ 40	0.821	0.3902	4.430	2.27	1.06–4.88	0.035
> 40	-	-	-	Reference	-	-
**Professional category**						
Nursing	0.421	0.4240	0.988	1.52	0.66–3.50	0.320
Medicine	-	-	-	Reference	-	-
**Years working**						
Over 11	0.381	0.3757	1.029	1.46	0.70–3.06	0.310
Up to 11	-	-	-	Reference	-	-
**Knowledge level**						
Adequate	−0.510	0.4569	1.246	0.60	0.25–1.47	0.264
Inadequate	-	-	-	Reference	-	-
**Intercept**	−1.204	0.6278	3.681	0.30	0.09–1.03	0.055

ICU, intensive care unit; CI, confidence interval.

## Discussion

This study demonstrated significant improvements in healthcare professionals’ knowledge and self-reported confidence immediately following a structured tracheostomy educational programme. Significant improvements were observed in knowledge of tracheostomy indications, bedside care, recognition of tracheostomy-related complications and overall knowledge scores following the intervention. These findings suggest that structured educational programmes may help address important knowledge gaps among healthcare professionals involved in tracheostomy care. Maintaining competence in tracheostomy care is particularly important because safe management of artificial airways requires adherence to established clinical principles and evidence-based care practices.^17^

Baseline knowledge varied across the different domains of tracheostomy care, with particularly low levels of knowledge observed for recognition of a blocked tracheostomy tube, recommended suctioning intervals for established tracheostomies and the management of tracheostomy-related complications. Previous studies from Pakistan, Ethiopia, Saudi Arabia, Türkiye, India and Sudan have similarly identified gaps in healthcare professionals’ knowledge of routine tracheostomy care and emergency management.^6,7,8,9,10,11^ However, direct comparison of overall knowledge levels across studies should be interpreted cautiously because assessment tools, scoring methods and thresholds used to define adequate or satisfactory knowledge differ between studies. Following the educational programme, significant improvements were observed across almost all knowledge domains in the present study. Similar improvements following structured educational interventions have been reported by Yelverton et al., Koç Özkan and Karaca and Abdulrahman et al.^11,12,13^

Participants’ self-reported confidence in tracheostomy tube insertion also improved significantly following the educational programme. However, self-reported confidence does not necessarily equate to procedural competence or objectively demonstrated emergency preparedness. The observed improvement should therefore be interpreted as an increase in participants’ perceived confidence rather than evidence of improved clinical performance. Similar improvements in self-reported confidence following structured tracheostomy educational interventions have been reported previously.^11,12,13^ Future studies incorporating objective simulation-based or clinical skills assessments are needed to determine whether improvements in confidence translate into demonstrable procedural competence and emergency preparedness.

Multivariable analysis demonstrated that assessment following the structured tracheostomy educational programme was independently associated with adequate knowledge after adjustment for potential confounding variables, supporting a significant association between the intervention period and improved knowledge. Participants with more than 11 years of clinical experience were also significantly more likely to demonstrate adequate knowledge, suggesting that cumulative clinical exposure may contribute to tracheostomy-related knowledge. However, associations between clinical experience and tracheostomy knowledge have not been consistently demonstrated in previous studies. Adithya et al. found no statistically significant association between years of experience and tracheostomy knowledge scores, highlighting that clinical experience alone may not reliably predict knowledge and reinforcing the importance of structured continuing tracheostomy education across levels of experience.^15^

Participants aged 40 years or younger demonstrated significantly greater odds of reporting high confidence than those older than 40 years. However, years of clinical experience were not independently associated with confidence. The reasons for this age-related difference cannot be determined from the present study and require further investigation. The outcomes evaluated in this study correspond primarily to Levels 1 and 2 of Kirkpatrick’s evaluation framework, reflecting participants’ reactions (confidence) and learning (knowledge acquisition). The study did not assess behavioural change in clinical practice (Level 3) or patient outcomes (Level 4), which represent increasingly important indicators of educational effectiveness. Future studies should incorporate workplace-based assessments, clinical audits and patient-centred outcomes to determine whether improvements in knowledge translate into sustained improvements in clinical practice and patient safety.

These findings have implications for healthcare delivery in resource-limited settings. Previous research has shown that adherence to tracheostomy care guidelines may be influenced by institutional factors including access to training, clinical protocols, supervision and organisational support.^18^ Structured tracheostomy educational programmes should therefore be considered one component of broader quality-improvement strategies aimed at strengthening safe tracheostomy care. Incorporating regular tracheostomy education into continuing professional development programmes may strengthen workforce preparedness for routine and emergency tracheostomy care.

A major strength of this study is the inclusion of healthcare professionals from multiple clinical disciplines involved in tracheostomy care, reflecting the collaborative nature of tracheostomy management in routine clinical practice. The educational programme combined didactic teaching with practical demonstrations of different tracheostomy tube types and incorporated a low-cost paediatric tracheostomy simulation model to demonstrate emergency paediatric tracheostomy tube replacement. Similar low-cost tracheostomy trainers have previously been described in the literature, including the model reported by Bonduelle et al. Our model was developed using inexpensive, locally available materials to provide an accessible training option for resource-constrained settings. This simple and reproducible model offers a practical educational strategy for institutions with limited access to commercial simulation equipment and may facilitate wider implementation of tracheostomy training. The simulation model was designed to teach the principles of emergency tracheostomy tube replacement, including orientation of stay sutures and controlled reinsertion of the tube. It was not intended to replicate tissue biomechanics or procedural complications such as false passage formation. Rather, it served as a low-cost educational adjunct to reinforce key procedural concepts where access to high-fidelity simulation is limited.

Alignment of the educational programme with internationally recognised tracheostomy safety principles enhances the potential applicability of this intervention across different healthcare settings. Incorporating recommendations from organisations such as the Global Tracheostomy Collaborative and the National Tracheostomy Safety Project ensures that educational content reflects current best practice while allowing adaptation to resource-constrained environments. Previous studies have similarly demonstrated improvements in tracheostomy-related knowledge following structured educational interventions.^11,12,13^ The use of paired pre-intervention and post-intervention assessments together with multivariable generalised estimating equation analysis further strengthened the evaluation of the educational programme.

In South Africa, the Breatheasy Tracheostomy Programme has demonstrated the value of structured multidisciplinary tracheostomy education, standardised clinical protocols and ongoing quality improvement initiatives in improving patient safety. Although our intervention consisted of a single 3-h educational workshop rather than a longitudinal programme, it complements these principles by providing a feasible introductory educational intervention that can be integrated into broader institutional tracheostomy safety initiatives, particularly in resource-constrained settings.

This study has several limitations. First of all, the one-group pre-test–post-test design without a control group limits causal inference. Improvements observed following the intervention may have been influenced, in part, by testing effects, whereby exposure to the pre-intervention questionnaire may have increased participants’ familiarity with the questions or sensitised them to the educational content.^19^ Knowledge and confidence were assessed immediately after the intervention, precluding evaluation of long-term knowledge retention, skills retention or behavioural change. Knowledge decay following educational interventions is well recognised in medical education, and immediate post-test improvements may diminish over time without reinforcement. Future research should evaluate long-term knowledge retention, procedural competence and patient outcomes using repeated follow-up assessments. Incorporating refresher training and spaced educational interventions may help mitigate knowledge decay and improve sustained competency in tracheostomy care. Consequently, this study cannot determine whether improvements in knowledge and confidence were retained over time or whether refresher training would be required to maintain these gains. Finally, the study was conducted at a single tertiary academic hospital using convenience sampling, which may limit the generalisability of the findings to other healthcare settings.

Future studies should incorporate longitudinal follow-up to evaluate knowledge retention and the sustainability of educational effects over time, including the potential need for refresher training. Multicentre controlled studies incorporating objective assessments of procedural competence and clinical performance are warranted to evaluate the generalisability of structured tracheostomy educational programmes and their potential effects on patient outcomes. Further research should also assess the effectiveness of low-cost simulation models for paediatric and adult tracheostomy training in resource-constrained settings.

## Conclusion

A structured tracheostomy educational programme significantly improved healthcare professionals’ knowledge and self-reported confidence regarding routine and emergency tracheostomy care. These findings demonstrate the potential value of structured education in addressing important knowledge gaps among healthcare professionals involved in tracheostomy management. However, increased self-reported confidence should not be interpreted as evidence of improved procedural competence, which was not objectively assessed in this study. Incorporating regular structured tracheostomy education into continuing professional development may strengthen healthcare professionals’ preparedness for routine and emergency tracheostomy care, particularly in resource-limited settings.
